# Proactive and Integrated Management and Empowerment in Parkinson’s Disease protocol for a randomised controlled trial (PRIME-UK) to evaluate a new model of care

**DOI:** 10.1186/s13063-023-07084-8

**Published:** 2023-02-27

**Authors:** Fiona E. Lithander, Emma Tenison, Jan Ypinga, Angelika Halteren, Matthew D. Smith, Katherine Lloyd, Edward W. Richfield, Danielle E. Brazier, Mícheál Ó. Breasail, Agnes J. Smink, Chris Metcalfe, William Hollingworth, Bas Bloem, Marten Munneke, Yoav Ben-Shlomo, Sirwan K. L. Darweesh, Emily J. Henderson

**Affiliations:** 1 Population Health Sciences, Bristol Medical School, University of Bristol, Bristol, BS8 1NU UK; 2 Liggins Institute, University of Auckland, Auckland, 1142 New Zealand; 3 Department of Nutrition and Dietetics, University of Auckland, Auckland, 1142 New Zealand; 4 Department of Neurology, Donders Institute for Brain, Cognition and Behaviour, Radboud University Medical Centre, P.O. Box 9101, 6500 HB Nijmegen, The Netherlands; 5 North Bristol NHS Trust, Southmead Hospital, Westbury-on-Trym, Bristol, BS10 5NB UK; 6 Bristol Randomised Trials Collaboration, Bristol Trials Centre, University of Bristol, Bristol, BS8 2PS UK; 7 Royal United Hospitals Bath NHS Foundation Trust, Combe Park, Bath, BA1 3NG UK

**Keywords:** Parkinson disease, Parkinsonian disorders, Parkinsonism, Randomised controlled trial, Complex intervention, Research design, Clinical trial, Informed consent

## Abstract

**Background:**

People living with Parkinson’s disease experience progressive motor and non-motor symptoms, which negatively impact on health-related quality of life and can lead to an increased risk of hospitalisation. It is increasingly recognised that the current care models are not suitable for the needs of people with parkinsonism whose care needs evolve and change as the disease progresses. This trial aims to evaluate whether a complex and innovative model of integrated care will increase an individual’s ability to achieve their personal goals, have a positive impact on health and symptom burden and be more cost-effective when compared with usual care.

**Methods:**

This is a single-centre, randomised controlled trial where people with parkinsonism and their informal caregivers are randomised into one of two groups: either PRIME Parkinson multi-component model of care or usual care. Adults ≥18 years with a diagnosis of parkinsonism, able to provide informed consent or the availability of a close friend or relative to act as a personal consultee if capacity to do so is absent and living in the trial geographical area are eligible. Up to three caregivers per patient can also take part, must be ≥18 years, provide informal, unpaid care and able to give informed consent. The primary outcome measure is goal attainment, as measured using the Bangor Goal Setting Interview. The duration of enrolment is 24 months. The total recruitment target is *n*=214, and the main analyses will be intention to treat.

**Discussion:**

This trial tests whether a novel model of care improves health and disease-related metrics including goal attainment and decreases hospitalisations whilst being more cost-effective than the current usual care. Subject to successful implementation of this intervention within one centre, the PRIME Parkinson model of care could then be evaluated within a cluster-randomised trial at multiple centres.

## Administrative information

**Table:** 

Title {1}	Proactive and Integrated Management and Empowerment in Parkinson’s Disease protocol for a randomised controlled trial (PRIME-UK) to evaluate a new model of care
Trial registration {2}	Clinicaltrials.gov reference number: submitted awaiting assignment of reference number
Protocol version {3}	version 4, 17 August 2022
Funding {4}	Gatsby Foundation
Author details {5a}	Fiona E Lithander^1,6^, Emma Tenison^1^, Jan Ypinga^2^, Angelika Halteren^2^, Matthew Smith^1^, Katherine Lloyd^1^, Edward W Richfield^3^, Danielle E Brazier^1^, Mícheál Ó Breasail^1^, Agnes J Smink^2^, Chris Metcalfe^4^, William Hollingworth^1,4^, Bas Bloem^2^, Marten Munneke^2^, Yoav Ben-Shlomo^1^, Sirwan K.L Darweesh^2^, Emily J Henderson^1,5^
Affiliations	^1^Population Health Sciences, Bristol Medical School, University of Bristol, Bristol, UK, BS8 1NU; ^2^Radboud University Medical Centre; Donders Institute for Brain, Cognition and Behaviour; Department of Neurology; P.O. Box 9101, 6500 HB, Nijmegen, The Netherlands; ^3^North Bristol NHS Trust, Southmead Hospital, Westbury-on-Trym, Bristol, UK, BS10 5NB; ^4^Bristol Randomised Trials Collaboration, Bristol Trials Centre, University of Bristol, Bristol, UK, BS8 2PS, ^5^Royal United Hospitals Bath NHS Foundation Trust, Combe Park, Bath, UK, BA1 3NG, ^6^ Liggins Institute and Department of Nutrition and Dietetics, University of Auckland.
**Name and contact information for the trial sponsor {5b}**	**University of Bristol Research and Enterprise Development, One Cathedral Square, Bristol, UK, BS1 5DD,** research-governance@bristol.ac.uk
Role of sponsor {5c}	The sponsor (University of Bristol) and the funding body (Gatsby Foundation) had no involvement in study design, analyses, nor interpretation of the results.

## Introduction

### Background and rationale {6a}

Parkinson’s disease (PD), the most common cause of parkinsonism, is a chronic neurodegenerative disorder that affects approximately 1% of the UK population aged over 60 years [1]. PD disproportionately affects older adults and it is estimated that the global prevalence will increase in the coming decades, in part due to growth in the ageing population, in part also due to cumulative effects of toxic chemicals in our environment [2]. Although there is significant heterogeneity in both the symptoms and the rate of disease progression in people with PD, the care pathways that are in place are not tailored towards individual phenotype and needs [3, 4]. Current care systematically lacks continuity and is not patient-led, and issues that people with PD and their caregivers face are often identified too late and managed reactively, instead of taking a more proactive approach [5]. There is also a greater need amongst people with PD to self-manage where active monitoring of their own physical and psychological status could be undertaken and appropriate decisions made. Additionally, a more coordinated and integrated approach between the multidisciplinary team members that manage the patient is required [6].

Integrated models of care have been developed for other chronic conditions including diabetes and coronary heart disease. The evidence is clear that self-management and behaviour change programmes improve outcomes [7]. It is evident therefore that novel and innovative models need to be established and robustly tested in people with PD given the high and fast-growing prevalence of this condition. A new model, called PRIME Parkinson (Proactive and Integrated Management and Empowerment), has been developed and is designed to manage issues proactively, deliver cohesive, multidisciplinary care, and empower patients and their caregivers towards greater self-management [8, 9]. Whilst this approach seeks to tackle issues in the delivery of healthcare, there is uncertainty as to whether it improves outcomes for patients and their caregivers or if it is cost-effective within a UK setting. The aim of this randomised controlled trial (RCT) of a complex intervention [10] is to determine if the PRIME Parkinson model of care will augment an individual’s ability to achieve their personal goals, and positively impact health and wellbeing in people with parkinsonism and their caregivers. Attainment of personal goals has been chosen as the primary outcome measure to reflect the highly heterogenous population that are being targeted with this individualised treatment approach.

### Objectives {7}

1. To determine if PRIME Parkinson care can improve the primary outcome of goal attainment, and secondary outcomes (encompassing measures across multiple domains of health-related quality of life and symptom burden), decrease hospitalisations, and be cost-effective in people with parkinsonism when compared with usual care.
2. Through mixed methods process evaluation, to explore how and to what extent the intervention was implemented and how and why the intervention was or was not beneficial.
3. To determine the effect of PRIME Parkinson care versus usual care on those caring for, living with, or supporting a person with parkinsonism.

### Trial design {8}

A single-centre, randomised controlled trial of PRIME Parkinson care versus usual care.

## Methods: participants, interventions and outcomes

This protocol is reported in accordance with SPIRIT (Standard Protocol Items: Recommendations for Interventional Trials) guidance.

### Study setting {9}

This trial will be delivered at a single centre, the Research Institute for the Care of Older People, the Royal United Hospitals Bath NHS Foundation Trust, in South West England. Participants will be recruited from the local geographical catchment area.

### Eligibility criteria {10}

The eligibility criteria for trial participation are shown in Table 1. Where there is uncertainty about the diagnosis, a decision will be reached from discussion and consensus between the patient’s usual treating clinician and the PRIME trial team. Patients must be able to provide informed consent to participate or where unable to do, there must be availability of a close friend or relative to act as a personal consultee. We have designed this complex intervention trial to ensure inclusion of under-represented groups with particular regard for those with cognitive impairment or dementia in order to maximise the generalisability of the findings. Cognitive impairment is common in people with parkinsonism [11], and this group is often precluded from participating in clinical research [12, 13]. Recognising the physical and psychological negative impact that caregivers may experience [14, 15], up to three informal caregivers, per person with parkinsonism, will also participate (Table 1).

**Table 1:** Inclusion and exclusion criteria for patients and caregivers

Group	Criterion	Definition
**Patients, inclusion**	Age	≥ 18 years
	Diagnosis of parkinsonism	Includes idiopathic Parkinson’s disease, progressive supranuclear palsy, corticobasal degeneration, multisystem atrophy, dementia with Lewy Bodies, vascular parkinsonism or primary progressive freezing of gait
	Location	Be resident within the geographical catchment area of the trial site
	Willingness	Be willing to participate
**Patients, exclusion**	Cause of parkinsonism	Drug, infection or toxin induced parkinsonism
	Capacity	Lack capacity to participate and do not have anyone who can be a consultee to provide advice regarding the patient’s wishes and views
	Decision of clinician	Current medical, cognitive or psychosocial issue or co-enrolment in other study that, in the opinion of the site investigator, would interfere with adherence to study requirements
**Caregivers, inclusion**	Age	≥ 18 years
	Provision of care	Provide informal care or support for a patient with parkinsonism
	Willingness	Be willing to participate
	Capacity	Have the ability to provide informed consent to participate
**Caregivers, exclusion**	Formal care	Professional caregivers who are paid to deliver care
	Person with parkinsonism not participating	Unwillingness or ineligibility of person for whom they provide care

### Who will take informed consent {26a}

Written informed consent will be taken from the patient and their caregiver(s), if relevant, at the baseline visit (Visit 1) by a member of the trial team. If, prior to Visit 1, there is indication from speaking to the patient, caregiver or a family member that the patient may lack capacity to consent, a capacity assessment will be conducted by phone by a trained member of the trial team to assess their ability to make a decision about research participation according to the Mental Capacity Act 2005 [16]. If the patient is unable to provide informed consent to participate, a close family member or friend who can act as a personal consultee will be identified and the patient’s prior wishes will be explored. If it is their view that the patient would wish to take part, they will be asked to accompany the patient to Visit 1 to complete a consultee declaration form. The personal consultee does not need to remain for the entirety of Visit 1 if a different caregiver or supporter can attend with the patient. If Visit 1 is held remotely over the phone or by videocall, the patient and consultee, as appropriate, will be asked to complete the consent form and/or consultee declaration form prior to the virtual visit and return it to the trial team by post. Patients are randomised to the intervention or the control arm at Visit 1.

### Additional consent provisions for collection and use of participant data and biological specimens {26b}

No biological specimens will be collected for storage.

## Interventions

### Explanation for the choice of comparators {6b}

The two arms of the trial are:

1. Intervention arm—PRIME Parkinson model of care plus usual care
2. Control arm—usual care

### Intervention description {11a}

The intervention is the PRIME Parkinson model of care which will be delivered by a multidisciplinary team including trained research staff, doctors and allied health care professionals, such as physiotherapists, occupational therapists and others. Patients in the intervention arm will continue to be offered usual care including follow-up with their regular Parkinson’s specialist. Contacts may take place face-to-face, in a patient’s home or another suitable location, remotely by phone or videocall. The intervention is a multi-component model of care comprising four components as follows [9]:

#### Care management

Patients will be assigned a care manager who will co-ordinate care and facilitate cooperation between those involved in their care [6]. Patients, their caregivers, general practitioner and secondary care teams will also have access to a ‘single point of access’ phone number and the call will be triaged by the study team towards the most appropriate PRIME team member. At Visit 1, patients will take part in a goal-setting interview and will be provided with a Personalised Care Plan where their priorities, current concerns and plan for how to address the goals identified will be documented.

#### Empowerment of patients and caregivers

This component encourages self-management of the condition. Patients and their caregivers will be provided with and supported to access relevant resources. They will be invited to attend group education workshops on topics such as medication management and nutrition. They will be signposted towards existing resources including information booklets, referral to third sector organisations, peer support and befriending services. Although patients who receive usual care can continue to access existing resources directly, intervention patients and their caregivers will be proactively signposted towards those which are most relevant to their needs and phenotype [17].

#### Empowerment of healthcare professionals

The PRIME multidisciplinary care team will comprise a multidisciplinary team of clinicians. The core team are supported to develop specialist clinical knowledge and skills to augment the successful delivery, the success of which will be evaluated of the PRIME intervention [18].

#### Development of specific IT infrastructure

A bespoke secure IT platform will be utilised which allows the intervention team to document their contacts with, and management plan for participants, and enhance communication to aid the coordination of care. It will also be accessible to patients themselves, as well as their caregivers. Patients and caregivers will be able to access educational materials which are relevant to the patient’s and the caregiver’s needs.

#### Control arm—usual care

Patients allocated to the usual care arm will continue to receive their usual care which includes scheduled follow-up by a movement disorder clinician or Parkinson’s nurse specialist. This is generally every 6 months and alternates between the clinician and the nurse. They may also have access to non-specialist physiotherapy and occupational therapy input, and access to resources such as those provided by Parkinson’s UK.

### Criteria for discontinuing or modifying allocated interventions {11b}

A patient and/or caregiver can choose to engage or not in any of the offered interventions in the active arm. Regardless of engagement and compliance with either the intervention and/or the assessments, participants will be encouraged to complete trial-related assessments. Enrolled participants from either arm can withdraw from the trial at any stage without prejudicing their usual care. Data collected until the point of withdrawal will be retained and used in the final analysis.

### Strategies to improve adherence to interventions {11c}

Engagement with the intervention will be monitored using process measures relating to each of the components of the PRIME Parkinson model of care and the intervention iteratively changed to improve adherence. Process measures will include data on usage of the single point of access, including source of the call, outcome of the triage process and action taken by the team; attendance at group educational sessions and engagement with educational materials; attendance at, and duration of, multidisciplinary team meetings; and the frequency with which the personalised care plan is reviewed and updated. The intervention is individualised and designed in conjunction with people with parkinsonism to maximise adherence.

### Relevant concomitant care permitted or prohibited during the trial {11d}

Usual care will continue to be offered to those patients in the intervention arm.

### Provisions for post-trial care {30}

Patients in the intervention arm will return to usual care at the end of the 24-month intervention period. Patients and caregivers can receive compensation for travel costs only.

#### Public and patient involvement

Public and patient involvement (PPI) representatives were included in the developmental stages of this protocol. They continue to be integrated in the study through providing feedback on patient facing documents including the participant information brochures, consent forms and the personalised care plan.

### Outcomes {12}

The primary outcome for patients is goal attainment which will be measured prospectively at 3 monthly intervals using the Bangor Goal-Setting Interview [19]. The primary outcome measure for patients will be analysed whereby an overall, unweighted mean rating for attainment across goals will be calculated at each evaluation point by dividing the sum of the ratings for all goals set by the participant by the number of goals set. Goal attainment will be additionally analysed with goals weighted according to the importance ratings stated by the participant at the goal-setting interview conducted at Visit 1. The primary outcome for caregivers is care-related quality of life measure using the Carer Experience Scale [20]. Outcome measures for patients and caregivers are described in Tables 2 and 3, respectively. A separate qualitative study will be carried out to explore the experiences of patients and caregivers recruited to the trial, and of hospital staff involved in the care of people with PD alongside staff involved in the delivery of the intervention. This will be reported separately.

**Table 2:** Outcome measures for patients which will be measured at baseline, 12 and 24 months

Domain	Outcome	Measurement tool/method where one is available
**Primary outcome measure**	Goal attainment	Bangor Goal-Setting interview [19]
**Parkinson’s specific measures**	Parkinson’s disease assessment	MDS-Unified Parkinson’s Disease Rating Scale (MDS-UPDRS) [21]
	Non-motor symptom burden	MDS Non-Motor Rating Scale (MDS-NMS)* [22]
	Parkinson’s-related quality of life	Parkinson’s Disease Questionnaire (PDQ-39)* [23]
**Health**	Fear of falling	Iconographical Fall Efficacy Scale (ICON-FES)-short version [24]
	Global impression of change	Patients’ Global Impression of Change (PGIC) Clinician Global Impression of Change (CGI-I)
	Frailty	SHARE-FI 75+ (Phenotypic frailty tool) [25] Pictorial fit frail scale* [26] Clinical frailty scale [27]
	Sarcopenia	SARC-F* and *SARC*-CalF* [28]
	Malnutrition risk	Malnutrition Universal Screening Tool (MUST)
	Nutritional risk	Seniors in the community: risk Evaluation for Eating and Nutrition (SCREEN II)-14 item version* [29, 30]
	Delirium	4AT tool for delirium assessment [31]
**Physical performance**	Physical performance	Short physical performance battery (SPPB) Time up and go test (TUG)
	Physical activity	Incidental and Planned Exercise Questionnaire for the Usual week (IPEQ-WA)* [32]
	Endurance	Endurance measure (2-min walk test, 6-min walk for those who are sufficiently mobile)
	Gait	Single and dual task gait assessments
	Grip strength	Hand-held dynamometer
	Falls^#^	N/A
**Palliative measures**	Advance Care Plan data	N/A
	Palliative symptom burden	Edmonton Symptom Assessment System Scale for Parkinson’s Disease (ESAS-R-PD) [33] Palliative outcome score-symptoms-Parkinson’s Disease (POS-S-PD) [34]
	Presence of gold standard framework register	Hospice utilisation outside place of death
	Healthcare contacts with hospice and / or palliative care services	Use of anticipatory medication
**Social**	Loneliness/social isolation	3-item Revised-UCLA Loneliness Scale plus a single item direct measure of loneliness [35]
	Social participation	English Longitudinal Study of Ageing questions (ELSA)* [36]
	Perceived social support	Multidimensional scale of perceived social support [37]
	Coping strategy	BriefCOPE [38]
	Acceptance of illness	Acceptance of illness scale [39]
	Capability	ICEpop CAPability measure for Older people (ICECAP-O)* [40]
	Patient activation	Patient Activation Measure (PAM) [41]
**Economic measures**	Health-related quality of life	EuroQoL 5D-5L health status questionnaire (EQ-5D-5L)* [42]
	Mortality^#^	N/A
	Healthcare events (including elective & unplanned admissions, emergency department attendances, outpatient appointments, primary care contacts, investigations and prescriptions, discharge destination)	Captured from hospital and GP records and participant self-report
**Process measures**	Frequency of Parkinson’s follow-up and referral to, and review, by allied health professionals (intervention and control arms)	N/A
	Frequency and type of engagement with PRIME Parkinson care (intervention arm)	N/A
	Experience of holistic patient-centred care	Patient Assessment of Chronic Illness Care measure (PACIC) - 26 item [43, 44]

*=outcomes that will be completed by a representative who knows the patient well, where the patient cannot self-complete; #=outcome measures which will also be assessed at 3, 6, 9, 15, 18 and 21 months. The following parameters will be assessed at baseline and/or at 12/24 months but are not outcome measures per se: fracture risk using FRAX* [45] and/or QFracture* [46] with bone densitometry where required; habitual dietary intake using the European Prospective Investigation into Cancer Food frequency questionnaire* [47]; comorbidity using Cumulative Illness Rating Scale-Geriatrics [48]; cognition using Montreal Cognitive Assessment (MoCA) [49]. Place and date of death, where applicable, will also be captured

**Table 3:** Outcome measures for caregivers which will be measured at baseline, 12 and 24 months

Domain	Outcome	Measurement tool/method, where one is available
**Caregiver measures**	Care-related quality of life	Carer Experience Scale (primary outcome) [20]
	Caregiver quality of life (PD-specific)	Parkinson’s Disease Questionnaire for carers (PDQ-carer) [50]
	Caregiver burden	Zarit Burden Interview [51]
	Caregiver activation level	Patient Activation Measure 13 UK (CG-PAM) [41]
	Caregiver coping strategy	BriefCOPE [38]
	Care (including relationship to recipient, living with recipient, intensity of caring, duration of care duties, tasks of caring)	N/A
**Health**	Frailty	Survey of Health, Ageing, and Retirement in Europe (SHARE-FI) 75+ [25]
	Sarcopenia	SARC-F and *SARC*-*CalF* [28]
	Malnutrition risk	Malnutrition Universal Screening Tool (MUST)
	Nutritional risk	Seniors in the community: risk Evaluation for Eating and Nutrition (SCREEN II)-14 item version [29, 30]
**Performance**	Physical performance	Short physical performance battery (SPPB) Time up and go test (TUG)
	Physical activity	Incidental and Planned Exercise Questionnaire for the Usual week (IPEQ-WA) [32]
	Grip strength	Hand-held dynamometer
**Social**	Loneliness/social isolation	3-item Revised-UCLA Loneliness Scale plus a single item direct measure of loneliness [35]
**Economic measures**	Caregiver costs	The Caregiver Indirect and Informal Case Cost Assessment Questionnaire [52]
**Process measures**	Frequency and type of engagement with PRIME Parkinson care (intervention arm)	N/A

The following parameters will be assessed at baseline and/or at 12/24 months but are not outcome measures per se: fracture risk using FRAX* [45] and/or QFracture* [46]; habitual dietary intake using the European Prospective Investigation into Cancer Food frequency questionnaire* [47]

#### Rationale for primary outcome

Given the heterogeneity of parkinsonian conditions, goal attainment was chosen as the primary outcome measure because it is patient-centred, meaningful to participants and not focused on a single symptom or domain. Goal-based approaches have been used successfully in frail older adults [53] and people with multiple sclerosis [54]. The Bangor Goal-Setting Interview has been applied in a multicentre trial of cognitive rehabilitation in people with early-stage dementia and has been shown to be feasible for use in people with Parkinson’s dementia and dementia with Lewy Bodies [55, 56].

### Participant timeline {13}

The duration of the intervention is 24 months.

### Sample size {14}

In the GREAT trial, the mean score for goal attainment at baseline was approximately 3.5 (SD 1.6) [55]. To detect a standardised effect size of 0.5 between groups (regarded as a moderate effect size [57] with 90% power, we would need 85 in each group. To allow for 20% attrition, this sample size has been inflated to give a total sample size of 214, with 107 patients in each arm. In terms of caregivers, a previous study has estimated that up to 80% of people with moderate to advanced Parkinson’s have an informal caregiver [58]. If 80% of the 214 recruited patient participants have an informal caregiver and 70% of these agree to take part, this would result in 120 caregiver participants (60 in each arm). With this number of caregiver participants, we could detect a difference of half a standard deviation in the primary outcome measure with 80% power and at a 5% significance level. This is equivalent to a 9 point difference in the CES index score, based on Rand et al. who reported a mean CES index score of 68.70 with a standard deviation of 17.78 amongst carers of adults using social care support in England [59]. We will have slightly greater power because we will recruit up to 3 caregivers per patient, although these will not be independent observations

Patients will be recruited predominantly through three routes. The first is where they will have expressed written interest in hearing about further research on a consent form through participating in other research studies. Secondly, they may be identified by the trial team from current hospital admissions or clinic lists. Thirdly, they may hear about the trial through local advertising or on social media. Potential participants will be sent an invitation pack containing a letter of invitation, a Patient Information Brochure, a pre-paid reply slip and a consent form, which is for information only. It will also include the equivalent documents for a potential caregiver who may wish to take part. If the team has not heard from the potential participant within 2 weeks of posting the invitation pack to them, they will receive a phone call and all questions will be answered. Potential participants and caregivers who indicate that they are interested in taking part, either by phone or by posting back the reply slip(s), will receive an appointment letter for Visit 1 with the relevant questionnaire booklet or a link to electronic questionnaires, depending on their preference.

## Assignment of interventions

### Allocation sequence generation {16a}

A minimisation algorithm will be used to avoid imbalance of age and disease severity across arms at baseline. The algorithm will randomly allocate patients to each arm with each newly recruited patient having an 80% probability of being allocated to the arm which achieves the best balance of age and disease severity, and a 20% probability of being allocated to the other arm. The two criteria used to minimise will be age, dichotomised at the median age of participants of the PRIME cross-sectional study, and Hoehn and Yahr stage, categorised as stages 1–2, stages 3–4, and stage 5. In this way, atypical and cognitively impaired participants should be well balanced but we will of course examine this at baseline and if necessary adjust for these predictors in our models. In the long run, this minimisation process will be expected to achieve a 1:1 allocation ratio.

### Concealment mechanism {16b}

Patients will be randomised after eligibility and consent have been confirmed at Visit 1, using an online randomisation system (Sealed Envelope, London, UK).

### Implementation {16c}

A member of the trial team will log onto the online randomisation system, enter the minimisation variables and request randomisation. The online system will generate the allocation code which will be displayed on screen. The allocated arm will be recorded on the password-protected IT platform and the participant informed by letter of the result. Members of the trial team who are conducting the blinded assessments will not have access to the allocated arm on the password-protected IT platform.

## Assignment of interventions: blinding

### Who will be blinded {17a}

Only the assessors who make 3-monthly phone calls to the patients will be blinded. Patients will be encouraged not to disclose their allocation to the assessor during these phone calls.

### Procedure for unblinding if needed {17b}

Not applicable.

## Data collection and management

### Plans for assessment and collection of outcomes {18a}

**Fig. 1:**
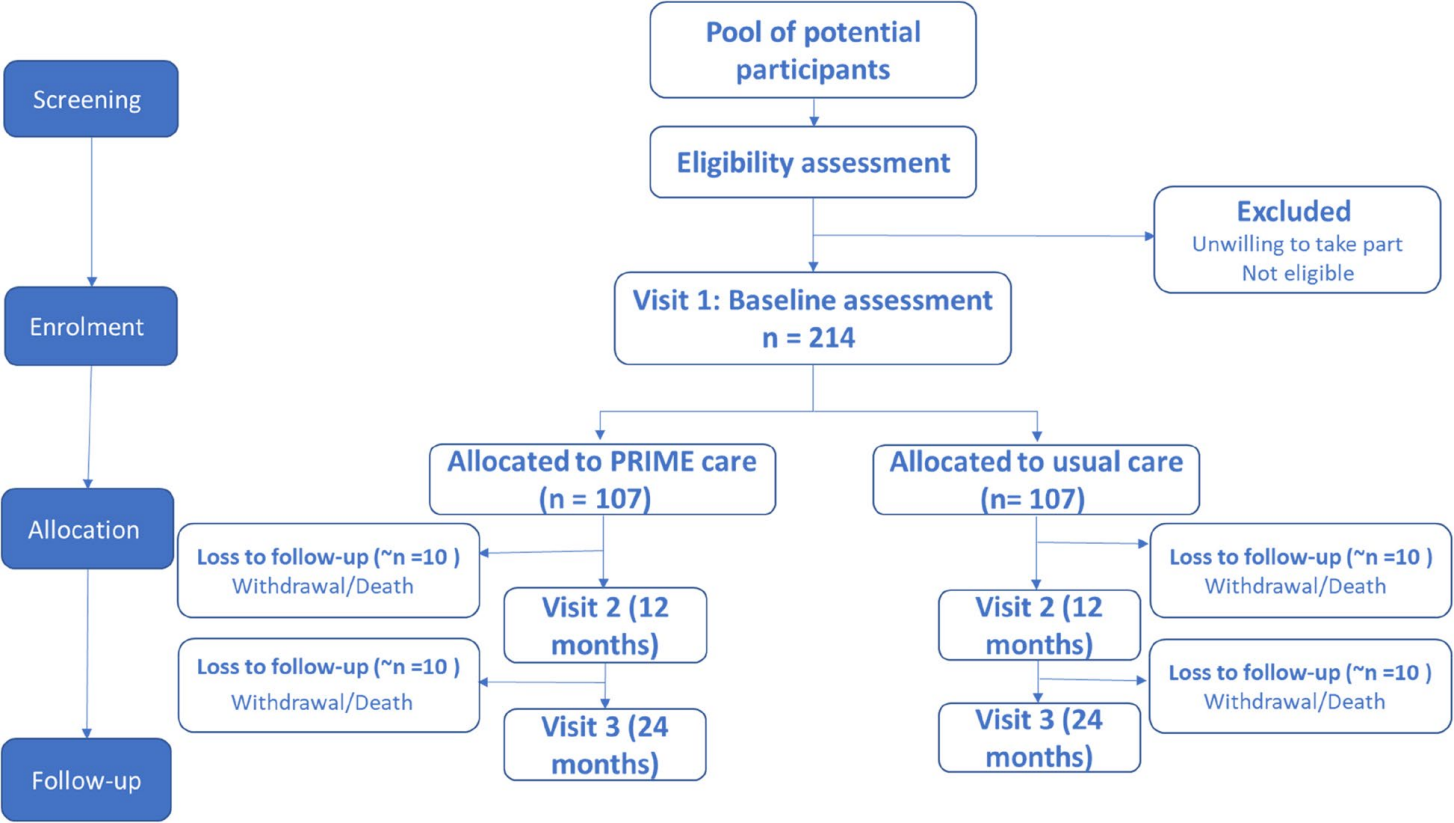
A flow diagram summarising participant flow through the study from screening through to follow-up

Data will be collected via participant-completed questionnaires on paper or electronically at Visit 1 (0 months), the mid-point assessment (12 months) and Visit 2 (24 months) and during the 3-monthly phone calls. Where a participating patient lacks capacity and is unable to complete the questionnaires, a representative will complete them on their behalf. The above methodology from screening through to follow-up is summarised in a flow diagram (see Fig. 1).

### Plans to promote participant retention and complete follow-up {18b}

Data on protocol violations, eligibility and recruitment rate will be reported. If randomised to the intervention arm, participants may opt out of interventions offered, but remain enrolled in the trial and therefore continue to complete questionnaires, undergo assessments and allow researchers access to their medical records. Patients, representatives where relevant and caregivers will receive phone or text message reminders to complete the questionnaires, depending on their preference.

### Data management {19}

When a participant consents to take part, they will be allocated a unique participant identification number. Consent forms and clinical letters with personal identifiable data, and completed paper questionnaires will be stored in a locked filing cabinet. Personal and research data entered directly onto the password-protected IT platform by participants or a member of the trial team and maintained within the University of Bristol will only be accessible to members of the trial team. Information capable of identifying participants will not be removed from the University of Bristol or the site nor will it be made available in any form to those outside the trial team, with the exception of National Health Service Digital for linkage to routine data. Participant details will be anonymised in all publications that result from the trial.

### Confidentiality {27}

The principles of confidentiality will be adhered to. Data will be collected and retained in accordance with the Caldicott Principles, UK Data Protection Act 2018 and General Data Protection Regulation. Personal data will not be kept for longer than is required. All data analysis will take place on encrypted, password-protected computers. No data will be released from the password-protected IT platform to any unauthorised third party without the written approval of Chief Investigator. Data will be available only for monitoring by the Research Ethics Committee or regulatory agencies. An archiving plan will be developed for all trial materials in accordance with the Sponsor’s archiving policy and trial materials will be archived for 5 years from the end of the trial.

### Plans for collection, laboratory evaluation and storage of biological specimens for genetic or molecular analysis in this trial/future use {33}

Not applicable. No biological samples will be collected as part of this trial.

## Statistical methods

### Statistical methods for primary and secondary outcomes {20a}

To determine if PRIME Parkinson care can improve goal attainment and impact the secondary outcomes, the primary analysis to determine whether PRIME Parkinson care can achieve goal attainment and improve health-related quality of life, symptom burden and hospitalisations will be conducted according to the intention to treat principle. Participants who provide primary outcome data will be included in the analysis in their allocated group (see below concerning missing outcome data). The intervention effect will be estimated as the coefficient of a binary covariate indicating treatment allocation in a linear regression with the goal attainment score as the outcome variable and with age and Hoehn and Yahr stage (minimisation variables) as additional covariates. We will examine the outcome at 24 months first, and if we observe no benefit, we will test if this was observed at 12 months. The estimated effect will be presented together with 95% confidence intervals and *p*-value. This approach to the analysis will be adapted to the participant secondary outcome measures, and to the outcome measures completed by caregivers. To determine the effect of PRIME Parkinson care versus usual care on those caring for, living with, or supporting a person with parkinsonism, robust standard errors will be employed to accommodate correlation between caregivers of the same participant.

### Interim analyses {21b}

No interim analyses are planned.

### Methods for additional analyses (e.g. subgroup analyses) {20b}

No subgroup analyses are planned.

We aim to determine whether the PRIME Parkinson intervention is cost-effective. Use of hospital, primary and community care will be ascertained from routine NHS data and participant self-report at follow-up and will be costed using national unit costs, where available. EQ5D-5L responses will be converted into utility scores using value sets recommended at the time of the analysis. Utility scores will be combined with mortality data to estimate quality adjusted life years. In the primary economic analysis, we will estimate the cost-effectiveness of the PRIME Parkinson intervention within the trial follow-up period from the perspective of NHS and social services. Based on national thresholds, we will calculate the net benefit for each patient and use net benefit regression to estimate the incremental net benefit (and 95% confidence intervals) and determine whether the PRIME intervention is cost-effective. In secondary analyses, we will expand the perspective of the analysis to include informal care costs, caregiver quality of life and patient wellbeing measures.

### Methods in analysis to handle protocol non-adherence and any statistical methods to handle missing data {20c}

Missing data will be imputed.

### Plans to give access to the full protocol, participant-level data and statistical code {31c}

Access to the data, and the statistical code used to derive and analyse the primary outcome measure, will be available through application to the Chief Investigator once the primary papers have been submitted. The statistical analyses plan will be made available prior to data analysis through a date stamped website.

## Oversight and monitoring

### Composition of the coordinating centre and trial steering committee {5d}

The Trial Management Group (TMG) meets every 4–6 weeks and is responsible for the day-to-day running of the trial. It is composed of the Chief Investigator and members of the trial team. The trial benefits from methodological input and governance from the Bristol Randomised Trials Collaboration which is a UK Clinical Research Collaboration Registered Clinical Trials Unit.

### Composition of the data monitoring committee, its role and reporting structure {21a}

The TMG reports to a joint Trial Steering Group/Data Management Committee (TSG/DMC) which meets at least annually with a remit to safeguard the interests of the trial participants, investigators and Sponsor, to assess the safety and efficacy of the trial’s interventions and to monitor the trial’s overall conduct. The membership of the TSG/DMC includes statisticians, a member of the Patient and Public Involvement Advisory Group, and an external clinician. In the event of the identification of a significant risk to participant safety, immediate measures would be taken which would include the suspension of recruitment and/or pausing PRIME Parkinson care if advised to do so by the joint TSG /DMC and/or the Sponsor.

### Adverse event reporting and harms {22}

Serious and other adverse events will be reported in accordance with the Good Clinical Practice guidelines and the Sponsor’s Research Related Adverse Event Reporting Policy. Participant safety will be monitored by the TMG, Sponsor and the joint TSG/DMC. The Chief Investigator or delegate will categorise all adverse events according to the accepted definitions of seriousness, expectedness and relatedness. Events expected within this patient population include hospitalisations, prolongation of hospitalisation or death which are probably related to parkinsonism; those related to, or a complication of a pre-existing health condition; hospitalisation for an elective surgical procedure, whether related or unrelated to parkinsonism, and expected side effects of the physical activity intervention including muscular discomfort. The site Principal Investigator is responsible for reporting all adverse events according to the Sponsor guidelines, and will report to the relevant committee as required.

### Frequency and plans for auditing trial conduct {23}

The Sponsor is responsible for monitoring the trial conduct.

### Plans for communicating important protocol amendments to relevant parties (e.g. trial participants, ethical committees) {25}

All protocol amendments will be approved by the Sponsor and the Research Ethics Committee, as relevant. The Chief Investigator or her delegate will highlight and disseminate changes to the trial team.

### Dissemination plans {31a}

Findings will be published in high-impact, peer reviewed, international journals, and presented at national and international conferences. Social media and the trial’s website will be used to disseminate progress and findings to all relevant stakeholder groups. Newsletters will be distributed to trial participants.

## Discussion

We have described a randomised controlled trial to investigate whether a unique and innovative model of care can improve the quality of life of people with parkinsonism and that of their caregivers in a single geographical area of the UK. Currently, there is equipoise as to whether new methods of care delivery will improve life for people with Parkinson’s in this UK setting [60]. It is also designed to investigate the cost-effectiveness of the intervention. Particular strengths of the trial are the inclusion of patients who lack capacity to consent, the inclusion of caregivers, the broad scope of parkinsonism that encompasses patients with dementia, rarer parkinsonian syndromes along with idiopathic disease, the longitudinal design that follows the participants for 24 months, the use of a wide range of outcome measures to capture the impact of the disease, and potential benefit of the intervention, holistically. These findings will complement those in the PRIME-NL study whereby a similar model of care is being evaluated in a prospective observational study delivered on a regional basis that focusses on regional collaboration [61]. In PRIME-NL, the primary outcome will be PD-related complications based on available proxies in healthcare claims data, whilst in PRIME-UK the primary outcome will be personal goal attainment. We aim to triangulate the results from both studies to provide greater insight on processes that may mediate any beneficial effects.

We recognise that the limitations include the restricted geographical area and the challenges of delivering and evaluating complex interventions [10]. This trial is an important early evaluation of the efficacy and cost of an approach designed to tackle pervasive issues in the delivery of health care for people with parkinsonism. We recognise that collecting data on goal attainment constitute an intervention in and of itself. This RCT is focused on learning how PRIME Parkinson can be delivered optimally to improve goal attainment whilst a future cluster RCT, where trusts will be randomised to implement the PRIME model or provide usual care is needed to provide stronger evidence of effectiveness [62].

### Trial status

The current protocol is version 4, dated 17 August 2022. Recruitment will commence in 2022 and will cease in 2023.

## Data Availability

Access to the data will be available through application to the Chief Investigator. Pseudo-anonymised data may be shared with other researchers to enable international prospective meta-analyses.

## Abbreviations

PRIME: Proactive and Integrated Management and Empowerment in Parkinson’s Disease
DMC: Data Monitoring Committee
PD: Parkinson’s disease
TMG: Trial management Group
TSG: Trial Steering Group
NL: Netherlands
RCT: Randomised controlled trial
